# Non-Standard T6 Heat Treatment of the Casting of the Combustion Engine Cylinder Head

**DOI:** 10.3390/ma13184114

**Published:** 2020-09-16

**Authors:** Jacek Pezda, Jan Jezierski

**Affiliations:** 1 Department of Manufacturing Technology and Automation, University of Bielsko-Biala, Willowa 2, 43-309 Bielsko-Biała, Poland; 2 Department of Foundry Engineering, Silesian University of Technology, Ul. Towarowa 7, 44-100 Gliwice, Poland; jan.jezierski@polsl.pl

**Keywords:** cylinder heads, heat treatment, Brinell hardness, automotive industry

## Abstract

The introduction of new design solutions of cast components to the powertrain systems of passenger cars has resulted in an increased demand for optimization of mechanical properties obtained during heat treatment, assuring—at the same time—a suitable level of production capacity and limitation of manufacturing costs. In this paper, research results concerning non-standard T6 heat treatment of a combustion engine cylinder head made of AlSi7Cu3Mg alloy are presented. It has been confirmed that the optimal process of heat treatment of this component, taking into consideration the criterion of material hardness, involves solutioning at a temperature of 500 °C for 1 h, and then aging for 2 h at 175 °C. As a result, HBS10/1000/30 hardness in the range of 105–130 was obtained, which means an increase from 35% to 60% in comparison to the as-cast, depending on the position of the measurement and spheroidization of precipitations of eutectic silicon.

## 1. Introduction

Aluminum and its alloys are used in various applications within the automotive industry. They are used for casting the majority of powertrain components of passenger cars, replacing cast iron in the applications such as cylinder blocks and cylinder heads, in order to reduce their mass.

Nearly 20% of CO_2_ emitted by humanity comes from transport. Reduction of emission of pollutants and consumption of energy, as well as the increase of recycling volumes [1], affect the natural environment and, in this aspect, the use of aluminum alloys in the automotive industry has become reasonable [2]. Due to economic and political pressure to reduce fuel consumption and CO_2_ emissions, engineering efforts aimed at reducing the mass of automotive structures and designs have been significantly strengthened [3,4], while in the recent decades, a specific engineering solution was developed, based on the intensive use of aluminum in the form of modified or new alloys [5,6,7,8,9], modern casting methods [10,11,12,13,14] and T6 heat treatment, involving solution heat treatment, water quenching, natural and artificial aging [15,16,17,18].

Production of molded automotive components (cylinder heads mostly) involves mainly the alloys from the 3xx.x series (Al-Si-Mg, Al-Si-Cu, Al-Si-Cu-Mg) [5,9,16,19,20]. Such alloys are characterized by perfect castability [10,14], relatively low mass (which affects the reduction of fuel consumption due to reduced total mass of a vehicle), as well as good mechanical [5,6,16,20,21] and technological properties. Nearly 100% of engine pistons, almost 75% of cylinder heads, 85% of exhaust manifolds and gearbox casings, as well as other power transmission elements (rear axles, differential housings, driveshafts etc.), are produced in the form of castings [2,5,22]. Aluminum castings are also used in chassis components, in up to 40% of wheels and mounts, components of brake systems, suspension and steering systems, as well as instrumentation dashboards [2,5,23,24].

A cylinder head made as a casting of silumin is one of the characteristic elements manufactured in the automotive industry. Mechanical and thermal loads impose special requirements on the structure of the cylinder head and, for this reason, the process of the casting of the cylinder head is very complicated. It also results from the fact that the geometry of the cylinder head is predominated by the system of working medium exchange, a system of cooling ducts, camshafts, and valves control mechanism incorporated inside. Moreover, the cylinder heads incorporate inlet and outlet valves, the layout and quantity of which (in case of 35 kW/L output, two valves are sufficient) are related to the shape of the combustion chamber and position of a spark plug in the center of the combustion chamber. Modern cylinder heads mounted in heavy-duty engines are equipped with even three, four, or five valves each, and possibly two spark plugs.

The cylinder heads, in the majority of the cases, are manufactured using the gravity casting process, poured into metal molds with their shape reflecting the external shape of the cylinder head, while inlet and outlet ports of the cooling system are modeled by sand cores positioned inside the molds [9,16,25]. Operational requirements imposed on heavy-duty cylinder heads, resulting from actual trends in engine development (downsizing), generate higher working temperatures and combustion pressures, enforcing the necessity of new technological solutions to assure strength and hardness in ambient and increased temperatures (up to 250 °C) [26,27] while maintaining high volumes of production.

The standard type of heat treatment performed according to the recommendations requires long-lasting solutioning and aging of the alloys. The ASTM B917-01 standard [28] is designated for the 319 alloys (with a chemical composition similar to the EN AC-AlSi7Cu3Mg alloy) cast into permanent molds and recommends up to 12 h of soaking at a temperature of 505 °C, hot water quenching, and then 2 to 5 h of aging at 155 °C, as recommended by the ASM Handbook [29]. On the other hand, 8 h of soaking at a temperature of 505 °C and aging for 2–5 h at temperatures between 150–155 °C is recommended by the Heat Treater’s Guide [30], while Zolotorevsky [31], in the case of the AK8M3 (AlSi8Cu3) alloy, advises heating at 500 °C for 5–7 h and aging at 180 °C for 5–10 h.

There are also publications pointing at the possibility of improving mechanical properties of aluminum alloys by even 30%, such as tensile strength, yield strength, and hardness, based on the heat treatment characterized by non-standard parameters of solutioning and aging operations [32,33,34,35].

In the paper, results of the research are presented, concerning the assessment of effects of shortened T6 heat treatment of the cylinder head obtained from a process of gravity casting, defined in terms of obtained Brinell Hardness (HB) hardness of the alloy on surfaces from the cylinder bores and camshafts side, as well as HB hardness in selected cross-sections.

## 2. Materials and Methods

AlSi7Cu3Mg alloy, belonging to the Al-Si-Cu-Mg group of alloys, is used, among others, to the production of castings of cylinder heads and cylinder blocks [9,36,37,38], as well as in other applications within the automotive industry, mainly in process of pouring into sand molds (engine crankcases, oil pans) and metal molds.

The investigated alloy of chemical composition as presented in Table 1, supplied directly by the manufacturer of the castings, was melted in an electric resistance furnace at a temperature of 720–760 °C.

Chemical analysis of the alloy was performed by using a method of optical emission spectrometry with inductively coupled plasma on the PerkinElmer optical emission spectrometer, model Optima 4300 Dv.

**Figure 1:**
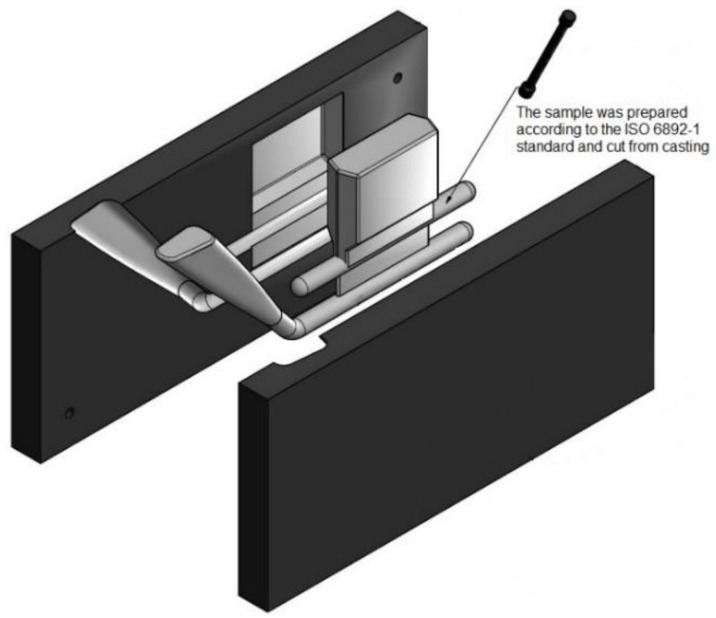
Metal mold for the pouring of the test pieces.

In the next step, permanent molds (Figure 1), purposed to the casting of the test samples used in static tensile tests, were poured with the investigated alloys. The temperature of the mold was kept at a constant level between 220–250 °C.

**Figure 2:**
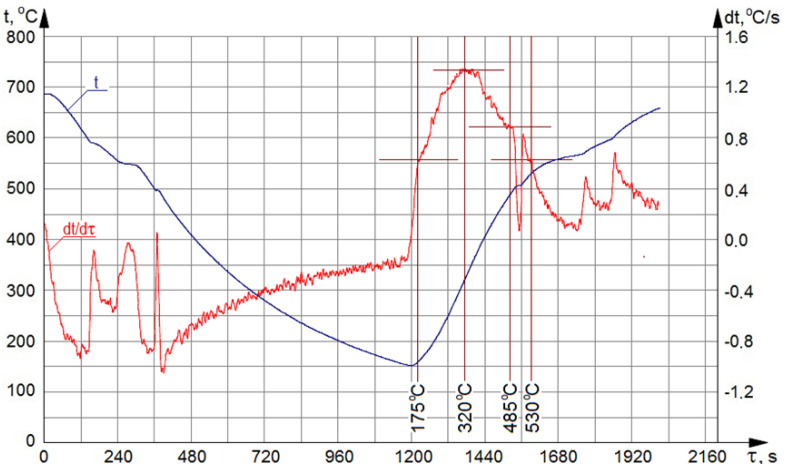
DTA curves for the investigated alloy.

The course of crystallization and heating processes, as well as the melting of the investigated alloy, with marked ranges of the solutioning and aging temperatures, are presented in Figure 2.

The DTA (derivative and thermal analysis) method involves continuous recording of the temperature of the alloy in course of its crystallization, which enables the generation of *t* = f(*τ*) curve, depicting the course of thermal processes. Simultaneously, a curve illustrating the derivative d*t*/d*τ*, highlighting less pronounced changes occurring on the thermal curve *t* = f(*τ*), is being plotted. Information obtained from the analysis of the course of the curves from the DTA method allows us to determine, with high accuracy, not only the melting temperature of a given alloy, but also melting temperatures present in an alloy of low-melting phases [39,40,41,42]. The solidification and melting process of the alloy were recorded with the use of a fully automated Crystaldimat analyzer, enabling measurement of the temperature of the test piece during solidification and melting. Standard DTA diagram is plotted during measurement for the process of solidification and melting of the alloy.

The heat treatment including solutioning operation, followed by the rapid cooling of the material in the water at a temperature of 20 °C (poured test pieces) and 20/60 °C (castings of the cylinder heads), had taken place; and then, artificial aging with cooling in the air.

The test pieces molded from AlSi7Cu3Mg alloy were soaked at temperatures of 485–530 °C and aged at temperatures of 175–320 °C. The time of the soaking operation amounted from 30 to 180 min, whereas the time of the aging operation amounted from 2 to 8 h. Basing on the assumed trivalent plan of the investigations with four variables (Table 2), heat treatment operations were performed for 27 systems (associations of temperature and time of solutioning and aging treatments).

Solutioning and aging operations of the test pieces and the castings were performed in a resistance furnace. Measurement of temperature was performed with the use of Ni-NiCr thermocouples of K type, with ±5 °C accuracy, directly in a chamber of the furnace, and concerned directly temperature of the test piece and the castings, temperature near heating elements (control system of the furnace), as well as temperature in the chamber of the furnace. Recordings of temperature in the chamber of the furnace and temperature of the test piece were performed continuously.

Heat treatment of the cylinder heads consisted of heating the casting in the furnace to solutioning temperature (500–515 °C), soaking at this temperature for 1 h, and then cooling in water (20 °C for cylinder heads I–III and 60 °C for cylinder head IV) and aging at temperature 175 °C for two hours.

Cylinder heads from the EN AC-AlSi7Cu3Mg alloy for the four-cylinder spark-ignition engine were produced in the technology of gravity casting into metal molds. Due to the restrictions imposed by the copyrights concerning the design of these cylinder heads, for the sake of this article, tested cylinder heads are presented in the form of drawings prepared based on 3D renders of the castings, made with the use of eviXscan Pro+ scanner, with an accuracy of 0.01–0.025 mm (according to VDI/VDE 2634 standard, part 2 [43]) and editable models from Geomagic Design X program.

**Figure 3:**
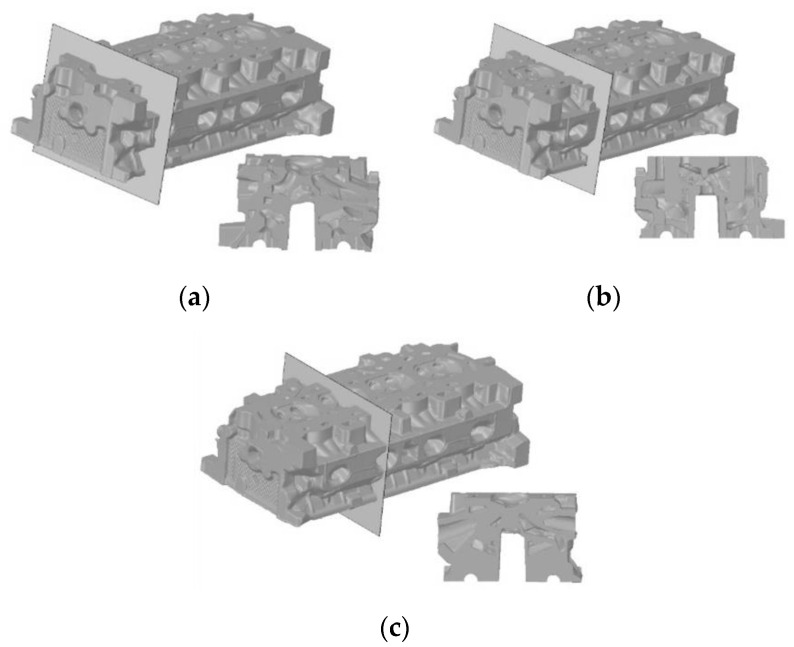
Surfaces for measurement of hardness of cylinder head casting: (**a**) cross-section 1 (55 mm from the face side), (**b**) cross-section 2 (115 mm from the face side), (**c**) cross-section 3 (160 mm from the face side).

**Figure 4:**
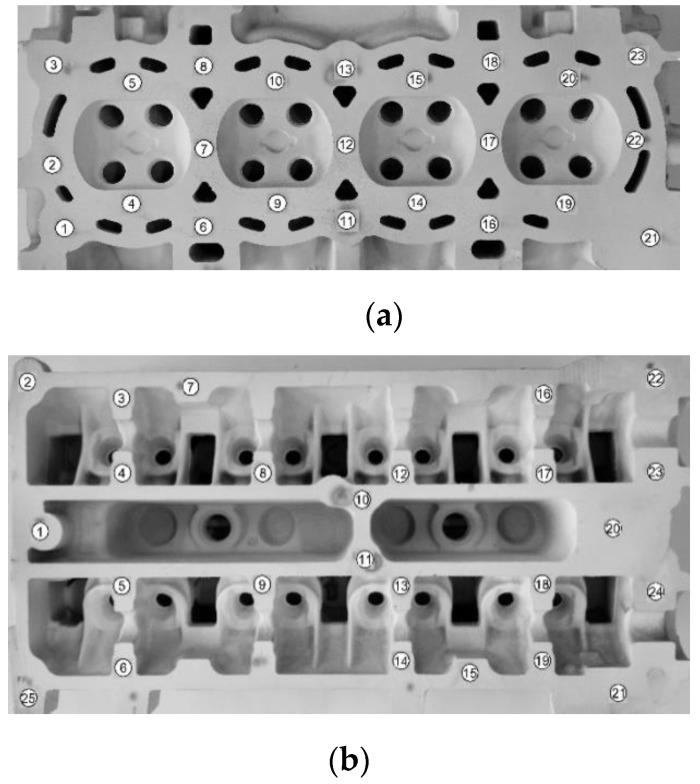
Positions of hardness measurement on milled surfaces of the cylinder head casting: (**a**) from the combustion chamber side, (**b**) from the camshafts’ side.

Measurement of the Brinell hardness was performed in compliance with the PN-EN ISO 6506-1:2014 standard [44] with the use of Brinell hardness tester of PRL 82 type, with a steel ball of 10 mm diameter, under 9800 N load sustained for 30 s. In the case of molded test pieces, the hardness was measured on milled heads of the test pieces; while in the case of the castings of cylinder heads—surfaces destined to measure the hardness, as shown in Figure 3 and Figure 4.

The HBS10/1000/30 hardness of the material was also measured on milled surfaces of the casting of the cylinder head from the combustion chamber side (Figure 4a) and the side of camshafts (Figure 4b).

“Statistica” ver. 13 software package, by StatSoft (Krakow, Poland), was used to write dependencies and plot diagrams showing the effect of heat treatment parameters on resultant hardness.

## 3. Results and Discussion

### 3.1. Heat Treatment of Cast Test Pieces

The HBS10/1000/30 hardness of raw (non-heat-treated) alloy was included within 82 ± 1.8 HB limits. Following heat treatment, the HBS10/1000/30 hardness of the investigated alloy amounted from 52 to 138.

**Figure 5:**
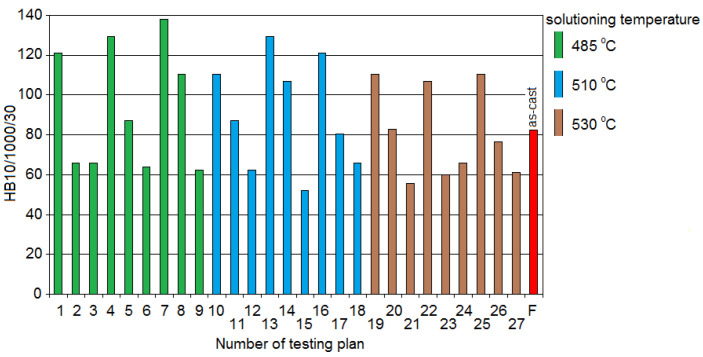
Effect of the heat treatment (of test samples) on the hardness of the AlSi7Cu3Mg alloy.

Making a comparison of the results of the raw alloy and the alloy after heat treatment (Figure 5), the highest increase of the hardness amounted to 138 HBS10/1000/30, confirmed for the system no. 7 (solutioning temperature 485 °C; solutioning time 180 min; aging temperature 175 °C; aging time 5 h), and hardness of 129 HBS10/1000/30 for the system no. 4 (solutioning temperature 485 °C; solutioning time 90 min; aging temperature 175 °C; aging time 8 h) and no. 13 (solutioning temperature 510 °C; solutioning time 90 min; aging temperature 175 °C; aging time 5 h). The test pieces from the systems identified by no. 1, 10, 19, and 25 were characterized by a slightly lower hardness (110–121 HBS10/1000/30), for which the aging temperature was equal to 175 °C. The lowest (within limits 52–62 HBS10/1000/30) hardness was obtained for the systems identified by no. 9, 15, 21, which were characterized by a high aging temperature (320 °C) for 8 h, which resulted in the reduction of obtained hardness concerning the raw casting.

Obtained results of performed investigations allowed us to formulate a dependency (1) in the form of a second-degree polynomial, depicting the effect of the heat treatment parameters on change of the HBS10/1000/30 hardness of the investigated alloy.

(1) $$\begin{array}{l} HB=-1044.73+5.519t_{s}-60.12\cdot 10^{-4}t_{s}^{2}+107.58\tau_{s}-0.43\tau_{s}^{2}-1.96t_{a}+8.58\cdot 10^{-4}t_{a}^{2}+7.73\tau_{a}-0.653\tau_{a}^{2}\\ \;\;\;\;\;-0.2t_{s}\tau_{s}+24.5\cdot 10^{-4}t_{s}t_{a}+0.002t_{s}\tau_{a}-0.01\tau_{s}t_{a}+0.338\tau_{s}\tau_{a}-0.02t_{a}\tau_{a} \end{array}$$

where: *t_s_*—solutioning temperature, *τ_s_*—solutioning time, *t_a_*—aging temperature, *τ_a_*—aging time. Correlation coefficient R^2^ = 0.96; correct. R^2^ = 0.9

**Figure 6:**
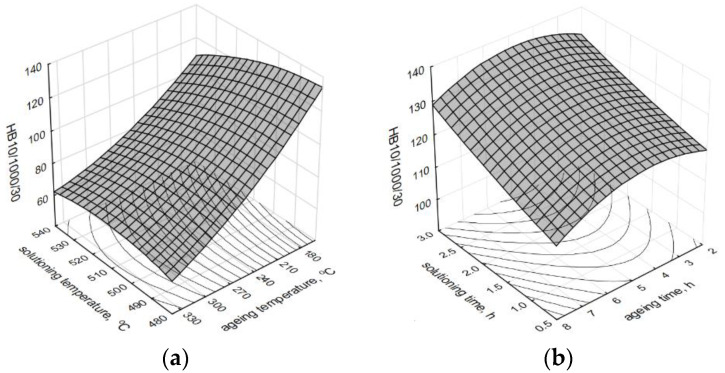
Influence of the heat treatment parameters on the hardness of the alloy. (**a**) *t*_s_ and *t*_a_, (**b**) *τ_s_* and *τ_a._*

Figure 6 presents spatial diagrams of the influence of temperature and time of solutioning and aging treatments on the HBS10/1000/30 hardness of the investigated alloy at preset parameters of the solutioning (*t*_s_—500 °C and *τ_s_—*1 h) and aging (*t*_a_—175 °C and *τ_a_—*2 h).

Taking into account obtained results of the measurements of the molded test pieces, it was ascertained that to obtain a considerable increase in the hardness, the alloy should be solutioned for 1–2 h at a temperature between 500–515 °C and aged for 2–5 h at a temperature below 180 °C (which should result in obtained HBS10/1000/60 hardness at the level of 120–130 (increase at the level of 50%)). Such hardness for the alloy of 319 brands with Mg additive (with a chemical composition similar to the investigated one) was obtained by the authors of the study [32] after solutioning at temperature 495 °C for 8 h and aging at temperature 170 °C for 4–8 h.

A similar range of solutioning temperatures (500–520 °C) was recommended by the author of the publication [45], while Han [46] took the temperature of 520 °C as an initial stage of the melting of Al_5_Mg_8_Cu_2_Si_6_ phase, dissolution of block-type precipitations of Al_2_Cu and spheroidization of precipitations of Si, with a direct effect on desired mechanical properties. According to Gauthier [47], solutioning temperature above 515 °C results in partial melting of copper phase on boundaries of grains; whereas, in the case of the alloy with 0.5% additive of Mg, Samuel [45] and Ouellet [48] note the beginning of partial melting of Al5Mg8Si6Cu2 and Al_2_Cu phases as early as at temperature 505 °C, resulting in the worsening of mechanical properties of the alloy. Górny [49] recommends the AlSi5Cu3Mg alloy to be solutioned at 510 °C for 5 h and aged for 10 h at temperature 170 °C, while the AlSi5Cu2 alloy at 490–495 °C and aged for 10–15 h at a temperature of 150–160 °C. A completely different approach, namely two-stage solutioning at temperature 495 °C for 2 h and at 515 °C for 4 h, followed by aging at temperature 250 °C for 3 h, resulting in an optimal combination of strength and ductility (HB > 98), compared to traditional single-stage solutioning at 495 °C for 8 h, was proposed by Sokolowsky in his study [33].

**Figure 7:**
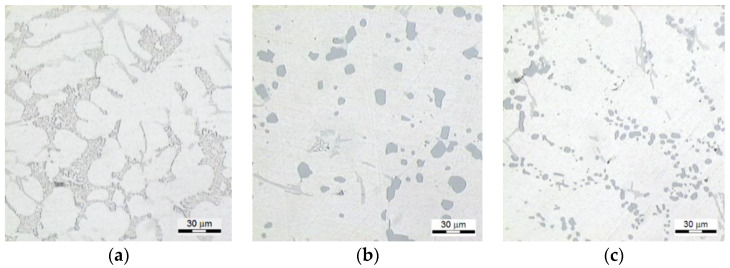
The microstructure of the alloy: (**a**) before the heat treatment—81 HBS, (**b**) for the system no. 27 (Table 2)—61 HBS, (**c**) for the system no. 13 (Table 2)—129 HBS.

In Figure 7, the microstructure of the investigated alloy before and after the proposed T6 heat treatment for selected systems from the investigations plan (Table 2) is presented.

Before the heat treatment, the microstructure of the alloy (Figure 7a) is characteristic of the eutectic Al + Si with small fibrous precipitations of Si and rounded contours of the plastic phase α(Al), which characterizes alloys after the modification [50,51]. The microstructure of the alloy after performed heat treatment, characteristic of the HBS10/1000/60 hardness at level 61 (Figure 7b) is characterized by distinctly coagulated large precipitations of Si. This is connected with a high temperature of solutioning (530 °C) and aging (320 °C), which promotes improved plasticity of the alloy with simultaneous reduction of mechanical properties, which is characteristic for the so-called over-aging of the alloy. However, the microstructure (Figure 7c) of the alloy after performed heat treatment and characteristic of high hardness (129 HBS10/1000/30) features precipitations of Si occurring in inter-dendritic areas of Al phase (on boundaries of grain), which feature rounded shapes and/or form of spheroidal precipitations.

### 3.2. Heat Treatment of the Cylinder Heads

In Table 3, the obtained ranges of the HB10/1000/30 hardness on the surface of the casting of the cylinder head (Figure 4) are presented.

Increase of hardness of the material of the cylinder heads marked by no. I–IV from 37 to 66%, compared to the raw casting, was obtained due to performed heat treatment operations. The difference in the hardness between analyzed surfaces results from the structure of the cylinder head’s material, which, in this case, is determined by the foundry method of the cylinder head (position of pouring risers from camshaft side) and, as in the case of the cylinder head no. IV, by a higher temperature of solutioning medium, limiting the rate of the cooling [52].

**Figure 8:**
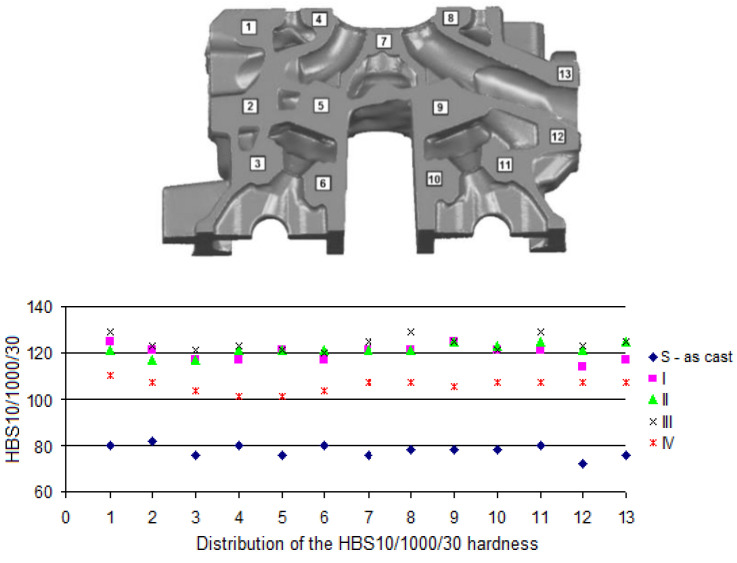
Areas of hardness measurement on the casting of the cylinder head—cross-section no. 1.

**Figure 9:**
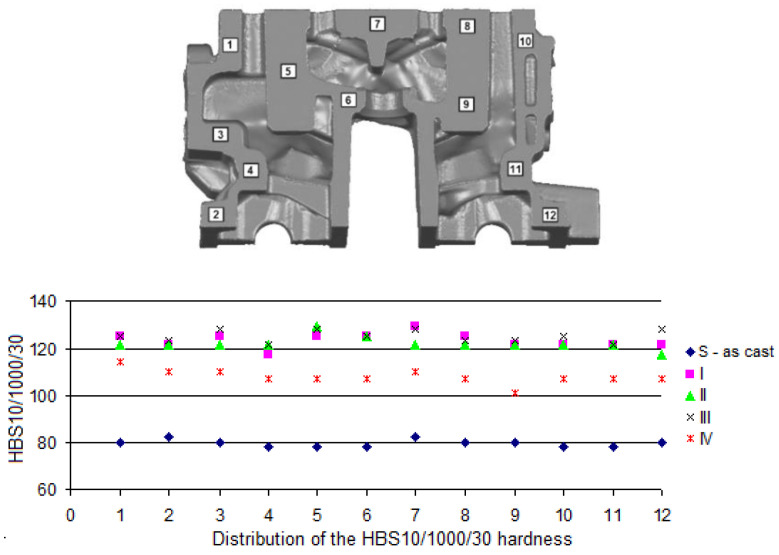
Areas of hardness measurement on the casting of the cylinder head—cross-section no. 2.

**Figure 10:**
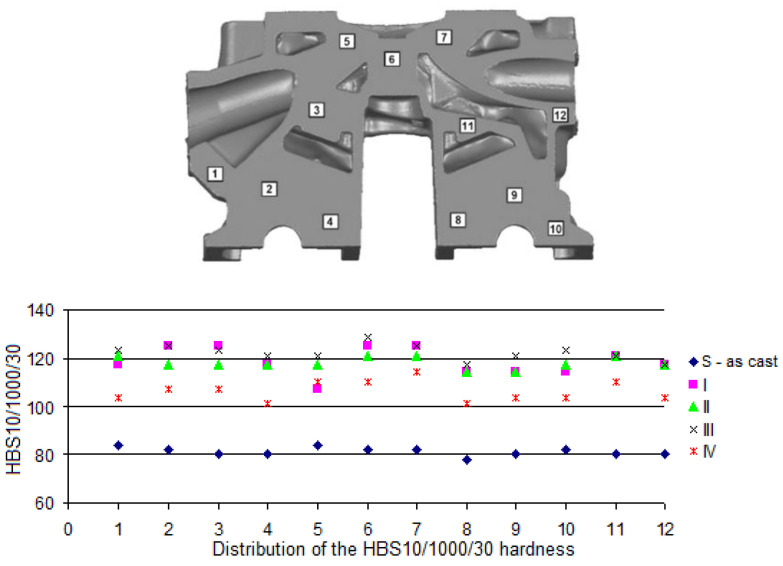
Areas of hardness measurement on the casting of the cylinder head—cross-section no. 3.

In Figure 8, Figure 9 and Figure 10, distributions of the HBS10/1000/30 hardness on selected cross-sections of the cylinder head (by Figure 3) are presented.

Obtained values of HBS10/1000/30 hardness on the cross-sections (Figure 8, Figure 9 and Figure 10) for the raw casting (without the heat treatment) were included within a range from 78 to 81. After performed heat treatment, the hardness was improved (increase to 100–130 HBS). The highest values of the HBS hardness can be seen in the locations where the highest cooling rate of the alloy occurs during crystallization, which has a positive effect on the results obtained after the heat treatment, which confirms the effect of the initial structure of the alloy on a run of hardening precipitation process [53,54], as well as the process of spheroidization of eutectic silicon, even though the 319.0 alloys are more resistant to the spheroidization, which can be attributed to lower solutioning temperature of 319 alloys (495 °C) in comparison with 356 alloys (540 °C) [55]. Attention should be paid to the fact that obtaining values of the hardness of the cylinder heads made of the AlSi7Cu3Mg alloy at the level of 101–107 HB required aging at 210–230 °C for 90–240 min (after solutioning at 498 °C for 120–480 min) [37], whereas a stable level of 135 HB was possible to be obtained after 24 h of solutioning of the casting at 485 °C and 5 h of aging at 180 °C [56].

**Figure 11:**
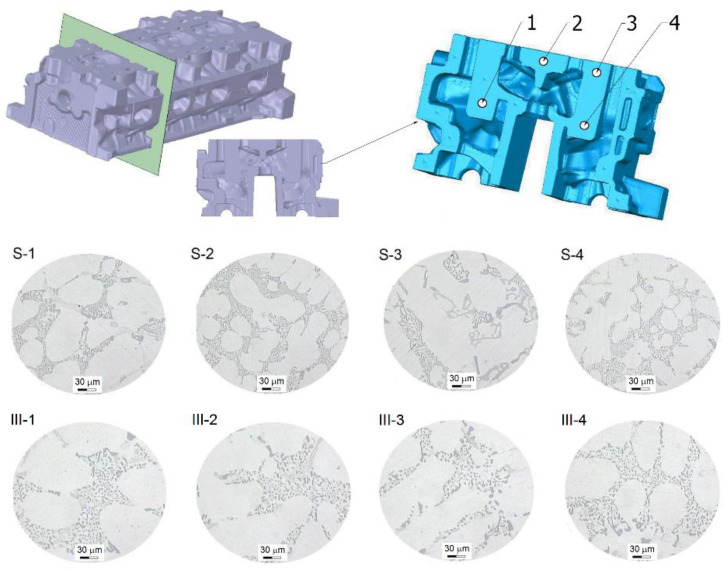
Microstructure in a selected area of the cross-sections of the cylinder head: S—the casting without heat treatment, III—the casting after the heat treatment (*t*_s_ = 500 °C, *τ*_s_ = 1 h, *t*_a_ = 175 °C, *τ*_a_ = 2 h).

In Figure 11, microstructures in selected areas of the cross-section no. 2 of the cylinder head casting before and after the heat treatment are shown.

The structure of the alloy in selected areas of the cross-section of the cylinder head (Figure 7) results from different cooling rates of the alloy during the crystallization process, hence, after the heat treatment, differences are also visible, mainly within the scope of the morphology of the precipitations of phase α(Al) and β(Si), in which precipitations are more dispersed in areas of its bigger crumbling in the eutectics α(Al) + β(Si) of the alloy before the heat treatment. Moreover, short soaking at a temperature of 500 °C, as well as aging at 175 °C has enabled obtaining a change in the form of the precipitations of silicon on the complete cross-section of the casting.

## 4. Conclusions

In terms of casting cylinder heads for combustion engines, it is possible to find such combination of the T6 heat treatment parameters (temperature, as well as the time of solutioning and aging treatments), which allows obtaining the highest value of HBS hardness with simultaneous consideration of the limited time of treatment operations.

Heat treatment performed on the test pieces poured from the alloy (from which the cylinder head of the combustion engine was produced) allows the determination of the tendency of the changes in the alloy’s hardness within the assumed range of the investigations.

Solutioning of the casting at a temperature of 500 °C for 1 h and then aging for 2 h at 175 °C has allowed obtaining HBS10/1000/30 hardness at the level of 105–130, which has assured its increase within limits 35–60%, in comparison to the raw casting.

Limiting the time of heat treatment of the combustion engine’s cylinder head, in case of a high volume of production of such type of casting, will allow an increase in the manufacturing capacity and decrease in the consumption of thermal energy.

## Figures and Tables

**Table 1:** Chemical composition of the investigated alloy (% wt.).

**AlSi7Cu3Mg**	**Si**	**Fe**	**Cu**	**Zn**	**Ti**	**Mn**	**Ni**	**Sn**	**Pb**	**Cr**	**Mg**	**Al**
	7.5	0.5	3.0	0.8	0.03	0.28	0.04	0.01	0.03	0.01	0.4	balance

**Table 2:** Investigations plan of the heat treatment of the EN AC-AlSi7Cu3Mg alloy.

Number of the System in the Experimental Plan	Solutioning		Aging	
	Temperature, °C	Time, h	Temperature, °C	Time, h
1	485	0.5	175	2
2			250	8
3			320	5
4		1.5	175	8
5			250	5
6			320	2
7		3	175	5
8			250	2
9			320	8
10	510	0.5	175	8
11			250	5
12			320	2
13		1.5	175	5
14			250	2
15			320	8
16		3	175	2
17			235	8
18			320	5
19	530	0.5	175	5
20			250	2
21			320	8
22		1.5	175	2
23			250	8
24			320	5
25		3	175	8
26			250	5
27			320	2

**Table 3:** HBS10/1000/30 hardness of the casting of the cylinder head.

Casting of the Cylinder Head	S (Raw Casting)	I	II	III	IV
Surface A *	76–84	129–138	125–129	129–138	107–114
Surface B *	72–78	117–129	110–114	121–129	101–106

* surface on the cylinder head as in Figure 4.

## References

[1] Kuchariková L., Tillová E., Bokůvka O. (2017). Recycling and properties of recycled aluminium alloys used in the transportation industry. Transp. Probl..

[2] Hirsch J., Hirsch J. (2014). Recent development in aluminium for automotive applications. Trans. Nonferr. Met. Soc. China.

[3] Pană G.M., Grigorie L.D. (2018). The Issue of Car Body Manufacture in Unibody Aluminum Alloy Design. Appl. Mech. Mater..

[4] Henriksson F., Johansen K. (2016). On Material Substitution in Automotive BIWs—From Steel to Aluminum Body Sides. Procedia CIRP.

[5] Padmanaban D.A., Kurien G. (2012). Silumins: The Automotive Alloys. Adv. Mater. Process..

[6] Peta K. (2017). Research on mechanical properties of aluminum alloys used in automotive industry. Inż. Mater..

[7] Pysz S., Maj M., Czekaj E. (2014). High-Strength Aluminium Alloys and Their Use in Foundry Industry of Nickel Superalloys. Arch. Foundry Eng..

[8] Shabani M.O., Mazahery A. (2014). Automotive copper and magnesium containing cast aluminium alloys: Report on the correlation between Yttrium modified microstructure and mechanical properties. Russ. J. Non Ferr. Met..

[9] Camicia G., Timelli G. (2016). Grain refinement of gravity die cast secondary AlSi7Cu3Mg alloys for automotive cylinder heads. Trans. Nonferr. Met. Soc. China.

[10] Kaufman J.G., Rooy E.L. (2004). Casting Properties, Processes, and Application.

[11] Szymanek M., Augustyn B., Kapinos D., Żelechowski J., Bigaj M. (2015). Al-Si-Re Alloys Cast by the Rapid Solidification Process/Stopy Al-Si-Re Odlewane Metodą Rapid Solidification. Arch. Met. Mater..

[12] Rosso M. (2012). Thixocasting and rheocasting technologies, improvements going on. J. Achiev. Mater. Manuf. Eng..

[13] Hong C.P., Kim J. (2006). Development of an Advanced Rheocasting Process and Its Applications. Solid State Phenom..

[14] Merchán M., Egizabal P., De Cortazar M.G., Irazustabarrena A., Galarraga H. (2018). Development of an Innovative Low Pressure Die Casting Process for Aluminum Powertrain and Structural Components. Adv. Eng. Mater..

[15] Akhtar M., Qamar S.Z., Muhammad M., Nadeem A. (2018). Optimum heat treatment of aluminum alloy used in manufacturing of automotive piston components. Mater. Manuf. Process..

[16] Molina R., Amalberto M., Rosso M. (2011). Mechanical characterization of aluminium alloys for high temperature applications Part 2: Al-Cu, Al-Mg alloys. Metall. Sci. Technol..

[17] Manente A., Timelli G. (2011). Optimizing the Heat Treatment Process of Cast Aluminium Alloys. Recent Trends in Processing and Degradation of Aluminium Alloys.

[18] Lumley R., O’Donnell R., Gunasegaram D., Givord M. (2007). Heat Treatment of High-Pressure Die Castings. Met. Mater. Trans. A.

[19] Fan K.L., He G., Liu X., Liu B., She M., Yuan Y., Yang Y., Lu Q. (2013). Tensile and fatigue properties of gravity casting aluminum alloys for engine cylinder heads. Mater. Sci. Eng. A.

[20] Molina R., Amalberto P., Rosso M. (2011). Mechanical characterization of aluminium alloys for high temperature applications Part 1: Al-Si-Cu alloys. Metal. Sci. Technol..

[21] Pezda J. (2014). Influence of heat treatment parameters on the mechanical properties of hypoeutectic Al-Si-Mg alloy. Metalurgija.

[22] Castella C. (2015). Self Hardening Aluminum Alloys for Automotive Applications. Ph.D. Thesis.

[23] Das S., Siddiqui R., Bartaria V. (2013). Evaluation of Aluminum Alloy Brake Drum for Automobile Application. IJSTR.

[24] Tocci M., Pola A., La Vecchia G., Modigell M. (2015). Characterization of a New Aluminium Alloy for the Production of Wheels by Hybrid Aluminium Forging. Procedia Eng..

[25] Köhler E., Klimesch C., Bechtle S., Stanchev S. (2010). Cylinder head production with gravity die casting. MTZ Worldw..

[26] Wang Q., Hess D., Yan X., Caron F. Evaluation of a New High Temperature Cast Aluminum for Cylinder Head Applications. In Proceedings of the 122nd Metalcasting Congress.

[27] Jeong C.-Y. (2013). High Temperature Mechanical Properties of Al–Si–Mg–(Cu) Alloys for Automotive Cylinder Heads. Mater. Trans..

[28] (2001). ASTM B917/B917M-01. Standard Practice for Heat Treatment of Aluminum-Alloy Castings from All Processes.

[29] (1991). ASM Handbook Volume 4: Heat Treating.

[30] Chandler H. (1996). Heat Treater’s Guide: Practices and Procedures for Nonferrous Alloys.

[31] Zolotorevsky V.S., Belov N.A., Glazoff M.V. (2007). Casting Aluminum Alloys.

[32] Tavitas-Medrano F.J., Mohamed A., Gruzleski J.E., Samuel F.H., Doty H.W. (2010). Precipitation-hardening in cast AL–Si–Cu–Mg alloys. J. Mater. Sci..

[33] Sokolowski J.H., Djurdjevic M.B., A Kierkus C., O Northwood D. (2001). Improvement of 319 aluminum alloy casting durability by high temperature solution treatment. J. Mater. Process. Technol..

[34] Zhang B.-R., Zhang L., Wang Z., Gao A. (2020). Achievement of High Strength and Ductility in Al–Si–Cu–Mg Alloys by Intermediate Phase Optimization in As-Cast and Heat Treatment Conditions. Materials.

[35] Pezda J. (2012). T6 Heat Treatment of Hypo-eutectic Silumins in Aspect of Improvement of Rm Tensile Strength. Arch. Foundry Eng..

[36] Javidani M., Larouche D. (2014). Application of cast Al–Si alloys in internal combustion engine components. Int. Mater. Rev..

[37] Chaudhury S.K., Apelian D., Meyer P., Massinon D., Morichon J. (2015). Fatigue Performance of Fluidized Bed Heat Treated 319 Alloy Diesel Cylinder Heads. Met. Mater. Trans. A.

[38] Torres R., Esparza J., Velasco E., Garcia-Luna S., Colás R. (2006). Characterisation of an aluminium engine block. Int. J. Microstruct. Mater. Prop..

[39] Szymczak T., Gumienny G., Wilk-Kolodziejczyk D., Pacyniak T. (2018). Effect of chromium on the crystallization process, microstructure and properties of hypoeutectic Al-Si alloy. Trans. Foundry Res. Inst..

[40] Smolarczyk P.E., Krupiński M. (2019). Thermal-Derivative Analysis and Precipitation Hardening of the Hypoeutectic Al-Si-Cu Alloys. Arch. Foundry Eng..

[41] Pietrowski S., Pisarek B. (2007). Computer-aided technology of melting high-quality metal alloys. Arch. Metall. Mater..

[42] Piątkowski J., Czerepak M. (2020). The Crystallization of the AlSi9 Alloy Designed for the Alfin Processing of Ring Supports in Engine Pistons. Arch. Foundry Eng..

[43] (2012). Optical 3D-Measuring Systems—Optical Systems Based on Area Scanning.

[44] ISO, 6506-1:2014 (2014). Metallic Materials—Brinell Hardness Test—Part 1: Test Method.

[45] Samuel F. (1998). Incipient melting of Al5Mg8Si6Cu2 and Al2Cu intermetallics in unmodified and strontium-modified Al–Si–Cu–Mg (319) alloys during solution heat treatment. J. Mater. Sci..

[46] Han Y., Samuel A., Doty H., Valtierra S., Samuel F.H. (2014). Optimizing the tensile properties of Al–Si–Cu–Mg 319-type alloys: Role of solution heat treatment. Mater. Des..

[47] Gauthier J., Louchez P.R., Samuel F.H. (1995). Heat treatment of 319.2 aluminium automotive alloy Part 1, Solution heat treatment. Cast Met..

[48] Ouellet P., Samuel F.H. (1999). Effect of Mg on the ageing behaviour of Al-Si-Cu 319 type aluminium casting alloys. J. Mater. Sci..

[49] Górny Z. (1992). Odlewnicze Stopy Metali Nieżelaznych.

[50] Dahle A., Nogita K., McDonald S.D., Dinnis C., Lu L. (2005). Eutectic modification and microstructure development in Al–Si Alloys. Mater. Sci. Eng. A.

[51] García-Hinojosa J. (2003). Structure and properties of Al–7Si–Ni and Al–7Si–Cu cast alloys nonmodified and modified with Sr. J. Mater. Process. Technol..

[52] Emadi D., Whiting L.V., Sahoo M., Sokolowski J.H., Burke P., Hart M. (2003). Optimal Heat Treatment of A356.2 Alloy. Light Metals-Warrendale-Proceedings.

[53] Magno I.A.B., Souza F., Barros A.D.S., Costa M.O., Nascimento J.M., Da Rocha O.F. (2017). Effect of the T6 Heat Treatment on Microhardness of a Directionally Solidified Aluminum-Based 319 Alloy. Mater. Res..

[54] Tavitas-Medrano F., Gruzleski J., Samuel F.H., Valtierra S., Doty H. (2008). Effect of Mg and Sr-modification on the mechanical properties of 319-type aluminum cast alloys subjected to artificial aging. Mater. Sci. Eng. A.

[55] Tash M., Samuel F.H., Mucciardi F., Doty H. (2007). Effect of metallurgical parameters on the hardness and microstructural characterization of as-cast and heat-treated 356 and 319 aluminum alloys. Mater. Sci. Eng. A.

[56] De Mori A., Timelli G., Berto F., Correia J.A.F.O., De Jesus A.M.P., Fernandez A.A., Calçada R. (2019). High Temperature Fatigue Behaviour of Secondary AlSi7Cu3Mg Alloys. Mechanical Fatigue of Metals: Experimental and Simulation Perspectives.

